# Climate change, risk perceptions and barriers to adaptation among forest growers in New Zealand

**DOI:** 10.1080/03036758.2023.2218103

**Published:** 2023-06-14

**Authors:** Grace B. Villamor, Steve J. Wakelin, Peter W. Clinton

**Affiliations:** a Scion (New Zealand Forest Research Institute, Ltd), Titokorangi Drive (formerly Longmile Rd), Rotorua, New Zealand; b Department of Ecology and Natural Resources Management, Centre for Development Research, University of Bonn, Bonn, Germany

**Keywords:** Actual adaptation decision, adaptation measures, risk perception, managed forests, subjective barriers

## Abstract

Climate change poses a significant challenge for forest growers. However, understanding climate change adaptation including the behaviour and decisions of forest growers remains unexplored in New Zealand, despite the forestry sector being a significant export leader and major contributor to regional economies. To explore this, we conducted surveys of 60 forest growers from organisations which collectively manage more than 70% of New Zealand’s plantation forests. The results showed that 47% of the respondents perceived that climate change will affect their forest growing, and 60% perceived that climate change will increase wildfire frequency and pest and disease outbreaks. Only 21% of the respondents reported that they had taken adaptation measures directly. Lack of resources and motivation were among the barriers that respondents identified. A logistic regression analysis revealed that climate change perception, research to improve forest growers’ adaptive capacity, climate change information, forest grower age and forestry experience are significantly associated with actual adaptation decisions. Risk perceptions central to protection motivation theory (e.g. vulnerability, probability and severity) were not linked to actual adaptation decisions, suggesting that widening adaptation analyses beyond individual perceptions as predictors of climate change adaptation may provide insights for fit-for-purpose climate change adaptation policies for the sector.

## Introduction

Climate change is one of the many critical issues that our forest growers face. If we want to minimise the risks associated with climate change and reduce the vulnerability of managed forests, adaptation is required (Nelson et al. 2016). Adaptation to climate change involves anticipating change, monitoring, and undertaking actions to avoid the negative consequences and to exploit the potential benefits of those changes (Keenan 2015). However, understanding New Zealand forest growers’ climate risk perception, their adaptation measures to reduce climate risks and barriers to adaptation remains unexplored, despite the sector being a significant export leader and major contributor to regional economies (Villamor et al. 2022). In New Zealand, 96% of plantation forests are privately owned and used for commercial timber production. These plantation forests provide tens of thousands of jobs with exports revenue worth approximately $6 billion annually (or 1.6% of the country’s GDP) (MPI 2022). Timber is the third-largest export earner behind dairy and meat. Thus, adaptation is crucial to reduce the risks to forest plantations, to protect the financial investments in forestry businesses, as well as to protect the sector’s contribution to the national economy, as chronic and acute hazards such as fire, pests and wind damage are projected to increase (Watt et al. 2019).

In this paper, we aim to understand risk perceptions of forest growers and the measures taken to minimise potential negative impacts. At the same time, this study serves as a basis for further work on risk communication, proactive risk management and formulation of forest policies related to climate change adaptation (Blennow et al. 2014; Nelson et al. 2016; Eriksson 2017). Moreover, understanding the risk perception of forest growers is crucial to evaluate their readiness to engage in adapting to climate change (Ameztegui et al. 2018).

Several factors may impede the implementation of adaptation decisions. These include subjective barriers (e.g. beliefs) resulting from cognitive and experiential processes (Blennow 2012) as well as structural barriers arising from broader economic, social or political conditions (Piya et al. 2019). Here, we consider barriers as obstacles that can be overcome with factors such as concerted effort, creative management, change of thinking, prioritisation, and related shifts in resources, land use, and institutions (Moser and Ekstrom 2010). Descriptively, barriers are impediments that can stop, delay or divert the adaptation process. Since forest management decisions have decadal and centennial-scale repercussions for forest landscapes, it is pertinent to be aware of these barriers as early as possible to adjust and avoid signals leading to maladaptation. In this paper, we explore the following research questions:

1. What are the forest growers’ personal views as to the local effects of climate change on their forestry assets and management practices?
2. Are forest growers adapting? What factors influence their actual adaptation decisions?
3. What barriers may hinder the implementation of climate change adaptation?

Identifying the perceptions of forest growers can inform society as to forest growers’ level of knowledge and degree of concern for climate change impacts, their understanding of risk and vulnerability, and current impediments or barriers to adaptation.

### Literature review

Perceptions of climate change have been found to be better predictors of the intention of climate change adaptation than socio-demographic characteristics (Stedman 2004). Several studies on risk perception of private forest owners conducted in managed forests underscored its importance as opposed to a top-down, policy-driven perspective on climate change (Blennow et al. 2012; Blennow et al. 2014; Seidl et al. 2016; Eriksson 2017). Furthermore, these studies reveal differences in terms of perceptions and adaptation between countries. For example, results from a survey conducted in the US show that individuals who believe climate change is unlikely to happen are less likely to be concerned about climate change impacts and less likely to support climate adaptation (Singh et al. 2017). In the European context, using the survey results of 508 forest professionals, Blennow et al. (2020) found that strong belief in the local impacts of climate change on the forest was a prerequisite for decision-making favouring adaptation.

Since the risks associated with climate change stimulate feelings of fear and anxiety and are psychological in nature (Williamson and Nelson 2017), which influences attitude change and decision-making towards adaptive practices, we have adopted the protection motivation theory (PMT) (Rogers 1975) in this study. PMT is a widely used framework to understand triggers that individual forest growers use to appraise potential threats. It has been applied to understand the climate change adaptation behaviour of individuals such as farmers and landowners (Grothmann and Patt 2005; Le Dang et al. 2014; Bostrom et al. 2019; Fischer 2019; Regasa and Akirso 2019). This framework involves threat or risk, and coping appraisals. In this study, we focus on threat or risk appraisal as a combination of perceived severity (perceptions regarding the degree of harm), perceived vulnerability (perception regarding the chance that one will experience harm) and perceived probability (perception regarding the likelihood of harm). Risk appraisal also focuses on the sources of the threat along with those stated factors that can increase or decrease the likelihood of making protective or non-protective responses. According to this theory, individuals are more likely to protect themselves when they anticipate negative consequences. Perceived risk – the belief that one is vulnerable to a risk factor – is thought to be a significant predictor of self-protective behaviour (Mead et al., 2012). Vulturius et al. (2018) suggested that personal risk appraisal and belief about the connection between personal experience and climate change can explain the adaptation of private non-industrial forest owners in Sweden.

Most studies that have applied PMT have used it to understand the climate change adaptation intention or motivation of individuals rather than actual adaptation responses, which is seen as a research gap (Andersson et al. 2017; Rodríguez-Cruz and Niles 2021). Hence in this study, we explore whether these variables are also determinants of actual climate change adaptation of forest growers.

## Materials and methods

### Respondents

This study is based on both online and paper-based surveys carried out among forest growers (e.g. owners, managers and consultants) who managed forests between 3 March and 3 May 2021. We sent the online survey to a list of forest growers and practitioners who had previously signed up for information updates. To include the small-scale forest owners (those who may have more limited access to internet and online surveys), a paper-based questionnaire was distributed to participants of the New Zealand Farm Forestry Conference on 11 March 2021. A combined total of 60 respondents participated in the online and paper-based surveys, with a response rate of 28%.

### Questionnaire

The questions analysed in this study were part of a longer questionnaire (Villamor et al. 2023), and only questions relevant to this part of the study are described here. The protection motivation theory and related research were adopted when preparing the questionnaire and pre-tested for refinements and adjustments with the assistance of representatives from the NZ Farm Forestry Association, a private forest owner and a forest consultant. The questionnaire asked background questions such as age, gender, forestry/farming experience, education, and the size of forest holdings. The following are the themes of the questionnaire used for this study:

*Perception of local climate change effects*: The forest growers responded as to how climate change will affect their forest growing activities, increase damage from forest pests and diseases, increase the frequency and severity of rural wildfires, and change their lifestyle, on a 5-point Likert scale (1 = strongly agree, 5 = strongly disagree).

*Climate-related risks/threats*: Respondents were asked to rank which of the climate risks concerned them the most. Then, three items were used for this study based on the PMT risk coping elements: (1) perceived severity, on a 5-point Likert scale (1 = extremely severe, 5 = not at all severe); (2) perceived vulnerability, on a 5-point Likert scale (1 = definitely vulnerable, definitely not vulnerable); and (3) perceived probability, on a 5-point Likert scale (1 = very likely, 5 = very unlikely).

*Adapting to climate change*: The forest growers were asked if they had taken measures to adapt their forest to climate change, on a 5-point Likert scale (1 = Yes, directly, 5 = do not know).

*Adaptation barriers*: Four themes were explored to determine how the following barriers impede forest growers’ adaptation process: (1) no motivation/energy to deal with climate change; (2) no resources (e.g. money) to adapt to climate change; (3) no ability to deal with potential dangers of climate change; and (4) not enough evidence of climate change taking place to adapt, or denial of climate change, on a 5-point Likert scale (1 = strongly agree, 5 = strongly disagree).

*Adaptation enablers*: Three items were used for this topic: (1) information on climate-related risks and warnings from official authorities; (2) research on climate resilient trees; and (3) social influence such as friends, family and neighbours, on a 5-point Likert scale (1 = strongly agree, 5 = strongly disagree).

### Data analyses

Descriptive statistics were used to summarise the characteristics of the 60 respondents. Weighted averages of the number climate risks reported by respondents were used to determine the relative importance. This is a better technique for estimating values of variables or aggregating scores in various fields than conventional averaging (Hoke et al. 1984). In addition, a binary logistic regression was used to explore the factors associated with the actual adoption of adaptive measures (Escarcha et al. 2018; Tesfahun and Chawla 2020; Upadhaya and Arbuckle 2021). The stated actual adaptation to climate change was the dependent variable. As much as we wanted to test all the variables associated with PMT along with barriers and enabling variables, our number of observations limits us. To avoid overfitting, we followed the rule of thumb of one predictive variable for at least 10 observations (Harrell Jr et al. 1996; Hair et al. 2014). Table 1 lists potential exploratory variables and brief descriptions including the expected relationship for this analysis. We followed the procedure devised by Rothman (2012, p. 223) in identifying relevant and important predictors based on the strength of the relationship to the outcome. Accordingly, the relationship is identified by quantifying the amount of change in the coefficient of the variable of interest (e.g. age) after each predictor variable is introduced in succession. If the coefficient of the variable of interest changes considerably (such as a change of greater than 10%), the predictor variable is added to the model as a confounder. This procedure was useful for identifying essential predictors while avoiding overfitting. Logistic regression results were presented in terms of an odds ratio as a measure of association between a variable of interest and the outcome. The larger the odds ratio, the more likely the association of the variable of interest is found with the outcome (Tenny and Hoffman 2017). If the predictor variable has an odds ratio of less than 1, it is less likely that the association of the variable of interest is to be found with the outcome. It is also important to look at the confidence interval for the odds ratio. If the odds ratio confidence interval includes 1, then it does not reach statistical significance (*p* < 0.05) (Grant 2014).

**Table 1.** List of potential exploratory variables and description.

Exploratory variable	Description	Expected sign
*Socio-demographic:*		
Age	Categorical (1 = under 18, 5 = 65+)	+/−
Forestry/farm experience (# of years)	Number of years in forestry/farm practice or operations	+
*Perception of local climate change effects:*		
*Perception 1*: My growing forests will be affected by climate change	Categorical, (1 = strongly agree, 5 = strongly disagree)	+
*Perception 2*: Climate change will lead to an increase in forest pests and diseases		+
*Perception 3*: Climate change will reduce tree growth/productivity		+
*Perception 4*: Climate change will increase the frequency and severity of rural wildfires		+
*Perception 5*: Climate change will change my lifestyle		+
*Risk perception:*		
Perceived severity	Categorical, (1 = extremely severe, 5 = not at all severe)	+
Perceived vulnerability	Categorical, (1 = definitely vulnerable, 5 = not at all vulnerable)	+
Perceived probability	Categorical, (1 = very likely, 5 = very unlikely)	+
*Barriers:*		
Denial of climate-related risk	Categorical, (1 = strongly	−
Lack of ability	agree, 5 = strongly disagree)	−
Lack of resources		−
Lack of motivation		−
*Enablers:*		
More research for improving adaptive capacity	Categorical, (1 = strongly agree, 5 = strongly disagree)	+
Climate change information and warning from official authorities		+
Social influence		+/−

## Results

### Descriptive statistics

Of the total of 60 forest growers who responded to the survey, 30% are forest managers managing on average of 63,000 ha of plantation forests; 30% are sole forest owners, who own an average of 2,000 ha of managed forests; 23% are forest consultants, who work for small- and large-scale forest companies; 12% have combined roles of forest owners, managers and consultants; and the remaining 5% are farmers who planted trees on their farms and/or recently harvested their trees. The respondents represent 90% of all the country’s regions. Around 87% of the respondents are male and 13% are female. Thirty-three per cent of the respondents are from the 55–66 year old age bracket, 23% are from the 45–55 year old age bracket, and 22% are older than 68 years of age. The respondents have an estimated average forestry growing experience of 29 years.

### Perception of local climate change effects

**Figure 1.**
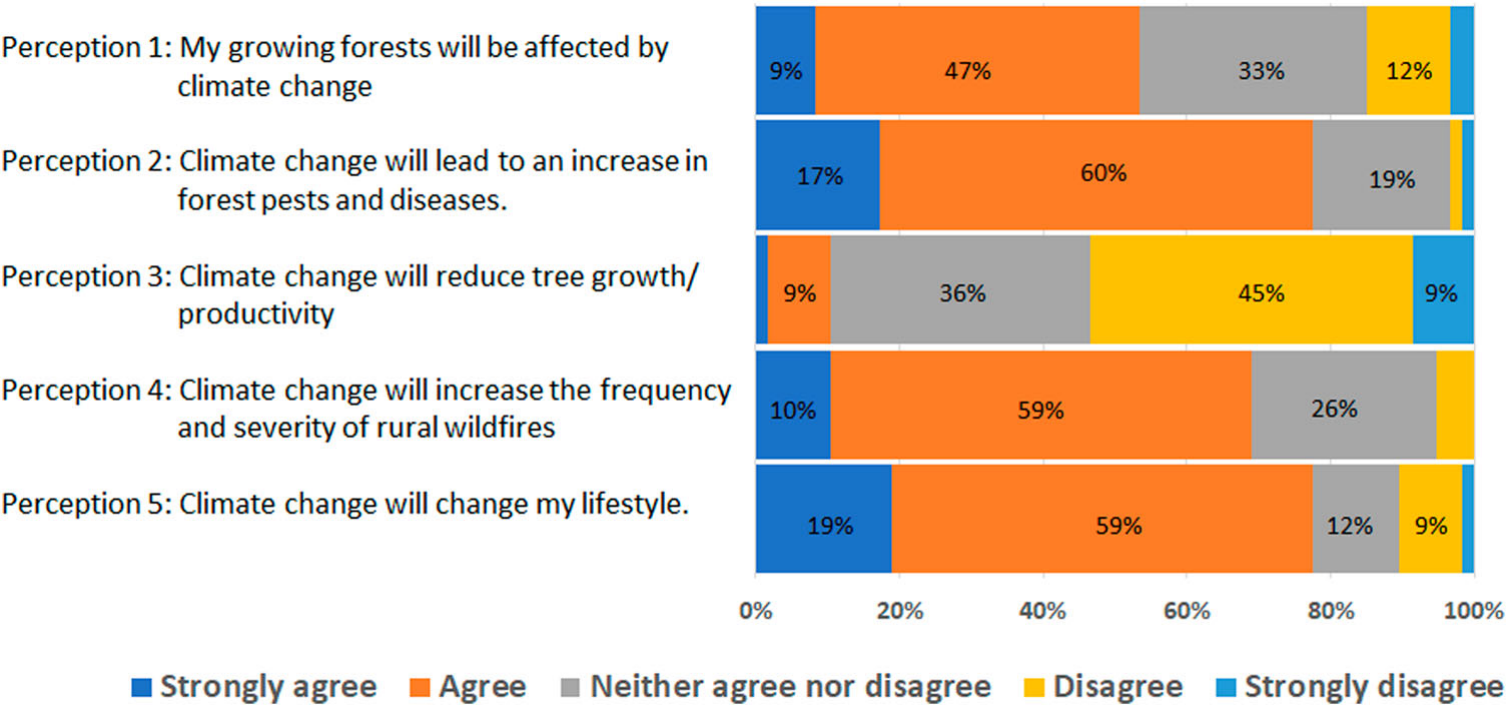
Perception of local climate change effects on forest growers.

Respondents agree that climate change will affect the growth of their forests (56%), increase the impact of pests and disease (77%), and increase the frequency and severity of wildfires (69%) (Figure 1). In contrast, 54% of the respondents disagree that climate change will reduce tree growth or productivity.

**Figure 2.**
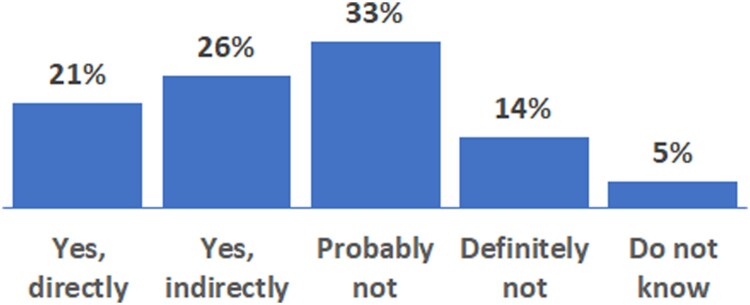
Percentage of respondents adapting to climate change (n = 55).

### Climate change-related risks

The risks that the respondents were most concerned with based on the weighted average of their ranking against other risks are presented in Table 2. Wind damage ranked first as the most worrisome climate-related risk to forest growers, followed by market risk. The majority of those who identified wind damage are forest owners and farmers, whereas market disruption due to climate change is mainly reported by forest managers and forest consultants. Other climate-related risks that respondents identified include drought and changing policies.

**Table 2.** Climate-related risks/threats as perceived by forest growers (n = 60).

Risk/threat	Weighted average	Mean ^2	Standard dev.	Ranking of importance (1 = most important)
Wind damage	2.63	8.56	2.44	1
Market risks due to climate change	2.77	10.55	2.79	2
Heavy rains, flooding, debris flows, landslides	3.26	12.60	3.06	3
Pest and diseases outbreaks	3.38	13.38	3.16	4
Forest fires/wildfires	3.43	14.02	3.25	5
Others	4.91	27.09	4.71	6

### Risk perception

Table 3 presents the risk appraisal on pest and disease outbreaks as perceived by respondents (which was used for perceived severity). Respondents anticipated both moderately (40%) and somewhat (40%) severe pest/disease outbreaks. Almost half of the respondents perceived that they are possibly vulnerable to pest and disease outbreaks that will physically affect their forests. With perceived probability, 48% of the respondents believed that pest and/or disease outbreaks will likely happen in the next five years.

**Table 3.** Pest and disease outbreak appraisal as perceived by respondents (n = 58).

Component	Response option	Respondents’ answer %
*Severity:* How severe do respondents anticipate a pest or disease outbreak could be?	Extremely severe	9
	Moderately severe	40
	Somewhat severe	40
	Slightly severe	9
	Not at all severe	2
*Vulnerability*: How vulnerable do you feel about the possibility of a pest or disease outbreak physically affecting your forest?	Definitely vulnerable	17
	Probably vulnerable	21
	Possibly vulnerable	46
	Probably not vulnerable	9
	Definitely not vulnerable	7
*Probability*: How likely is it that a pest or diseases outbreak will happen in the next five years?	Very likely	10
	Likely	48
	Neither likely nor unlikely	24
	Unlikely	17
	Very unlikely	1

### Adaptation measures/strategies

When the respondents were asked whether they had taken any measures to adapt to potential climate change impacts on their forests, only 21% of them reported taking direct measures (Figure 2).

Table 4 lists the measures or actions planned by respondents to reduce or manage each of their reported climate-related risks. Across all these risks, the frequently reported measures include silvicultural activities (e.g. flexible harvesting schedule), planting mix and use of resistant tree species (e.g. *Pseudotsuga menziesii* – resistant to insect borer, wind and snow damage; *Cupressus macrocarpa* – tolerant to exposed site conditions; *Sequoia sempervirens* – fire resistance; *Eucalyptus delegatensis* – tolerate frosts of −9°C), training, information, and preparedness. Measures specific to particular risks were reported such as market diversification for addressing market risk, and biosecurity surveillance for addressing pest and disease outbreaks.

**Table 4.** Proposed adaptation measures according to specific risk.

Risk/threat	Proposed adaptation measures/strategies
Wind damage	Timing of operations (e.g. sequencing/flexible harvest schedule; timely thinning operations) Planting with fast-growing species as shelterbelts Insurance Planting wind-tolerant species
Market risk	Market diversification (both domestic and export) Diversify forest Establish resilient genetics Labour supply and cost management Flexible harvesting schedule Improve awareness and information
Heavy rains, landslides	Retiring high-risk slopes Flexible harvesting schedule Improving ground cover Genetic improvement Establishing erosion control measures Improving forest engineering design and standards
Pest/disease outbreak	Growing mix of tree species; diverse trees and genetics Planting resistant varieties (i.e. genetics) Biosecurity surveillance Biosecurity training and research Preventive measures for visitors
Wildfire outbreak	Fire breaks establishment, pruning activities Insurance Planting fire-resistant species Fire preparedness and response plan, training & equipment Redesigning plantation layout Training and close relationship with FENZ
Others (drought, policy-related risks)	Increase rates of contractors Short rotation Stronger career pathways (e.g. from labour to forest management position) Increase training, education, and communication with future local force

### Adaptation barriers and enablers

**Figure 3.**
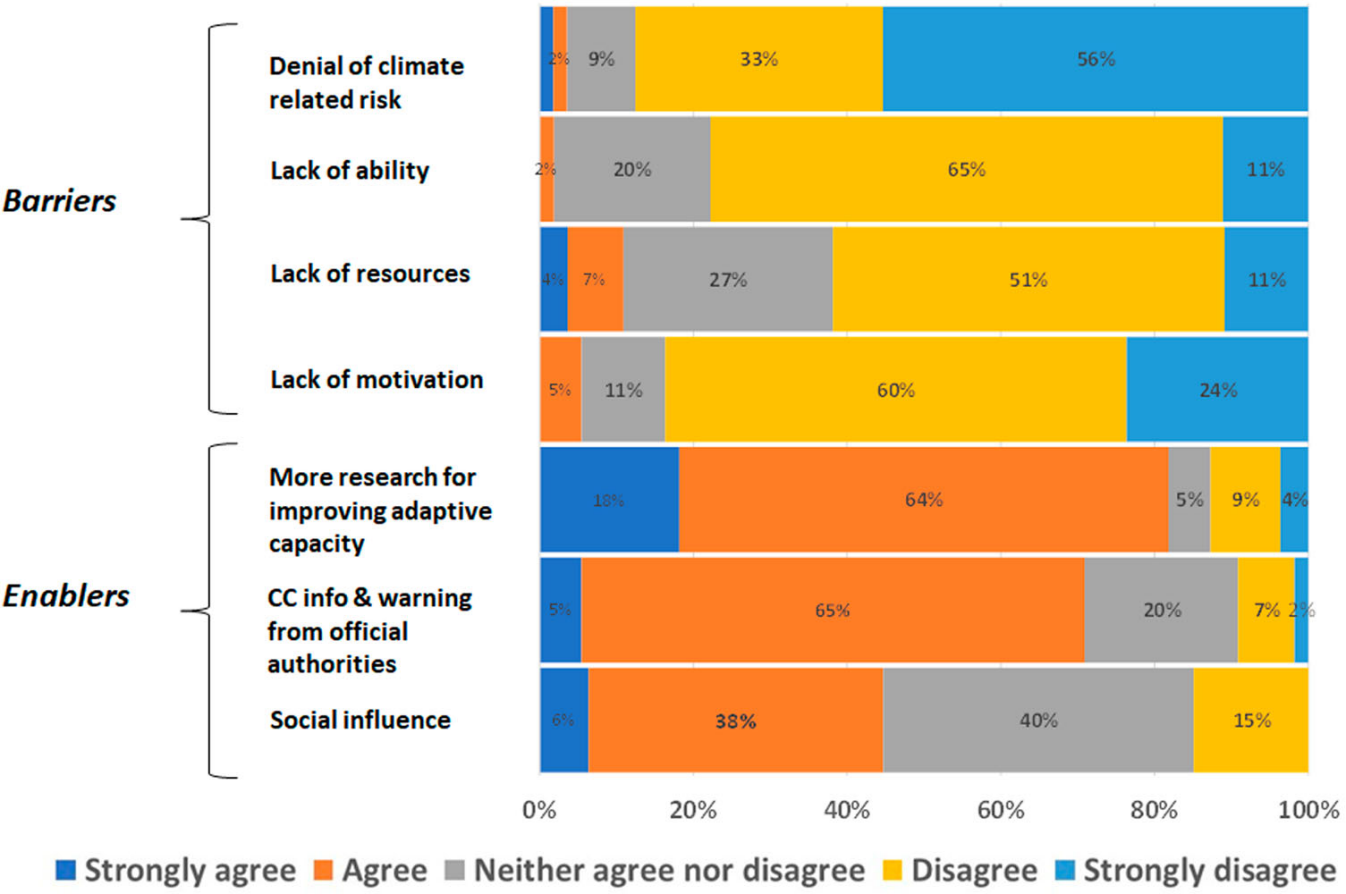
Percentage of respondents’ perception regarding barriers and enablers of adaptation.

Figure 3 illustrates the barriers to and enablers for adaptation as perceived by the respondents. In terms of barriers to adaptation, the majority of the respondents disagreed that they lack ability (65%), resources (51%) and motivation (60%); whereas 56% of the respondents strongly disagreed that they perceive themselves as climate deniers.

In contrast, 4% of the respondents strongly agreed with lack of resources as a barrier to adaptation. For enablers, 82% agreed or strongly agreed that more research on climate change will improve their adaptive capacity. In addition, 65% of respondents agreed that climate-related risks information and warning from official authorities will improve their capacity to adapt to climate change. Respondents had more mixed responses in terms of social influence. For example, a high proportion (40%) neither agreed nor disagreed that social influence was an enabler of adaptation and there were few with strong opinions either way.

### Factors affecting actual adaptation decisions

**Figure 4.**
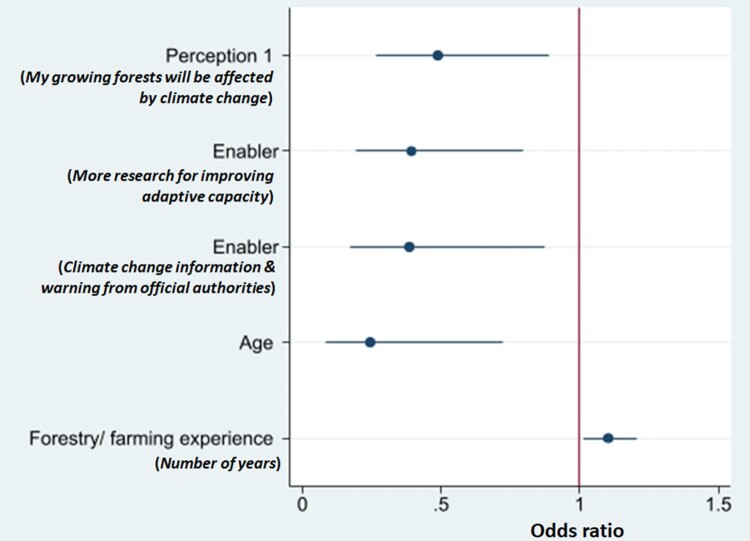
Odds ratio of the variables in a binary logistic regression model of adopting an adaptation strategy to reduce climate change impacts to their forest growing (R^2^ = 0.32; *p* < 0.0003; n = 52).

Figure 4 shows the factors associated with the actual adaptation decisions. All the variables are significantly (*p* < 0.05) associated with the actual adaptation decision of forest growers. Only *forestry experience* has an odds ratio of more than 1, which means that with each additional year of experience in forestry or farming, the odds or likelihood of adopting an adaptation measure increases by 10%. This suggests that the more experience the forest grower has with forestry and farming, the greater the odds of adopting an adaptation measure. On the other hand, four of the variables have odds ratios less than 1, namely:

- Each increase of unit of scale (i.e. going from strongly agree to strongly disagree) for the *perception that their growing of forests will be affected by climate change* is associated with a 52% decrease in the odds or likelihood of adopting adaptation measures. This suggests that those forest growers who adopted adaptation measures perceived that their forest growing will be affected by climate change.
- Each increase of the unit of scale (i.e. from strongly agree to strongly disagree) for *more research to improve the adaptive capacity of forest growers* is associated with a 61% decrease in the odds or likelihood of adopting adaptation measures. This suggests that forest growers who adopted adaptation measures agreed that more research is needed to improve their adaptive capacity.
- Each increase of the unit of scale (i.e. from strongly agree to strongly disagree) for *climate-related risks information and warning* is associated with a 62% decrease in the odds or likelihood of adopting adaptation measures. This suggests that those forest growers who adopted adaptation measures agreed that climate risk information and warning from official authorities will improve their adaptive capacity.
- Each increase of the unit of scale for *age* (from younger to older) is associated with a 76% decrease in the odds of adaptation decision. This suggests that the older the forest grower, the lower the odds or likelihood of adopting adaptation measures.

## Discussion

### What are the forest owners’ personal views about the local effects of climate change on their forests?

The survey results showed that almost 60% of the respondents perceive that climate change will lead to an increase in the occurrence and impact of forest pests and diseases on their forests, and also increase the frequency and severity of rural wildfires. Wind damage was the most frequently perceived climate change risk amongst forest growers. This is a similar finding to Sweden where intensive forestry is also a vital component of the national economy (Blennow 2012; Andersson et al. 2017). Private forest owners in Sweden also have the strong perception that climate change will substantially affect their forests.

Just over half of New Zealand’s forest growers also perceived that climate change would not reduce their forest productivity, which is consistent with previous modelling that indicates that climate impacts on productivity coupled with greater CO_2_ levels will result in increased productivity on average, before accounting for disturbances such as pests, disease and wildfires (Watt et al. 2019).

In terms of comparisons with New Zealand farmers, the 2018 farmers survey focusing on dairy, livestock and arable (MPI 2019) reported that 52% of farmers think their farms and business are moderately or majorly impacted by current climate or severe weather patterns, and this outlook has not changed since 2009. But the proportion of farmers reporting no impact at all has declined (19% to 10%). Around 59% anticipate a moderate or major impact over the next 20 years. Unfortunately, direct comparison is not possible because we focus on local effects on forest growing. However, we can gain a sense of similarity in terms of the percentage of responses (i.e. 56% of respondents agree that climate change will affect their forests in the future).

### Are forest growers adapting?

The answer is yes and no. In our study 46% of the respondents reported they had already directly and indirectly taken measures to adapt their forests to climate change (Figure 2). Frequently reported adaptation strategies were related to silvicultural responses (e.g. thinning operations, flexible harvesting) and diversification of tree species and markets. In comparison with the survey results of private forest owners who adapted their forest management to climate change in Sweden (20%), Portugal (47%) and Germany (54%), our finding is relatively comparable with the results from Portugal (Blennow et al. 2012).

### What factors influence their actual adaptation decisions? What barriers may hinder the implementation of climate change adaptation?

With regard to determinants of actual adaptation decisions, none of the PMT variables (e.g. perceived severity, vulnerability and probability) was associated with actual decisions, despite the perceived level of risk of climate change is between the moderately and somewhat risky levels. This may be due to the small sample size of this study (see discussion on study limitations). Findings from a recent study by Rodríguez-Cruz and Niles (2021) showed that neither perceived capacity nor motivation were linked to actual adaptation behaviours and further corroborated our findings. Instead, we found that acceptance or acknowledgement of the local effects of climate change was significantly associated with actual adaptation decisions. According to Blennow et al. (2012) and Yousefpour et al. (2013), beliefs and perceptions of the local effects of climate change have often been linked to more support for climate change actions.

Research that will improve an individuals’ adaptive capacity and provide new climate change-related information was also found to enable actual adaptation decisions. These results agree with the findings of Sousa-Silva et al. (2016), suggesting that improving the communication and demonstration of possible solutions for climate change adaptation will increase adaptive capacity, and this is therefore likely to be an effective strategy for increasing the adoption of adaptation measures. Also, our results on climate change information and research were consistent with a survey of climate issues of farmers in New Zealand that found that ‘*despite not having actively sought information, farmers express interest in further information or advice about improving resilience to climate change*’ (MPI 2019, p. 5). Furthermore, the report showed that farmers do not seek information directly from the government, but instead use as their main sources of climate information and advice industry events/field days (55%), rural professionals (53%) and industry organisations (48%), which probably include large-scale forest companies.

Our study found that socio-demographic variables such as age and forestry experience were significantly associated with actual adaptation, in contrast to findings from other studies (Blennow 2012). Although we did not ask participants to explain their reasons, these factors may suggest that they are not as interested in investing in adaptation if they expect to be retired well before the trees are mature and/or if they have already planted and pruning or thinning has taken place. This observation aligns with the findings of a report on farmers’ climate change adaptation (MPI 2019). Accordingly, because New Zealand’s agriculture sector has an ageing population, the levels and types of actions in relation to sustainable land measures vary by farmer age. For some farmers, taking on debt to improve the climate change resilience of their farms may be a less attractive option while others are looking ahead to their retirement.

In addition, such socio-demographic variables could also be seen as barriers for forest growers’ climate change adaptation. From the 2017 Survey of Rural Decision-Makers, the majority of the respondents (56%) who reported practising forestry activities belonged to the 56–85 age bracket (Brown 2017; Villamor et al. 2022). According to a report on forest owners’ intentions (Manley 2018), a lot of farm foresters are old and will delegate their decision-making to someone else. Like the farmers, most of the forest growers belonged to an ageing population, suggesting that adopting climate change adaptation strategies would be a challenge for current owners.

Many of the respondents in our study said they disagreed that lack of ability, motivation and resources are barriers to their implementation of climate change adaptation. Only about 7% of the respondents agreed that they lack resources, while 5% of respondents agreed that they lack motivation.

### Limitations

Because forest management system adaptation is a multi-scale incremental process (both temporal and spatial), the knowledge we gained from this study does not represent the many diverse actors in the forestry sector. For example, there are approximately 14,000 smallholder forest owners, who own and manage almost 30% of the total production forests (NZFOA 2018). These growers are not well represented in this study or in the 2018 survey of farmers on climate change issues (MPI 2019). They are a difficult group to engage (widely geographically distributed in rural areas with limited internet connections) plus their demographic is less likely to consider adaptation (NEFD 2019). However, this group does require further study to assess their adaptive capacity. Furthermore, we assumed that the risk perceptions of professional foresters working for forest companies differ from small-scale forest owners/operators, which we were unable to explore due to our small sample size, and we acknowledge it as a limitation. Nevertheless, this study provides a baseline for the current forest growers of the country and there are some similarities with New Zealand farmers surveyed on climate change issues (MPI 2019). Regarding our main dependent variable, we did not evaluate types of practices (e.g. if they are recommended for adaptation) or how other variables than those related to adaptation perceptions had direct effects on the actual adoption of risk reduction measures. Furthermore, we only focused on pest and diseases outbreak in detail in terms of perceived severity, vulnerability, and probability. Other climate-related risks such as windthrow, flooding and drought deserve to be further analysed.

## Conclusions

This study provides a baseline understanding of forest growers’ perceptions of climate change as well as potential barriers to climate change adaptation. A logistic regression model was used to examine the correlations of individual forest grower factors with the actual adoption of climate change adaptation measures. Socio-economic variables such as age and forestry experience were found to be significant in explaining their actual adaptation decisions.

Forest growers hold a range of beliefs and perceptions about climate change and there are similarities in our findings to findings from earlier studies in other countries. This suggests that strategies to encourage adaptation that has been successful elsewhere may be applicable in New Zealand.

Research and more information about climate change adaptation are likely to drive further adaptation. This effort should be targeted towards early career forest decision-makers and the older ones who are still actively planting and replanting forests. Given the increasing risk of climate change impacts, this should become a priority in helping forest growers contribute to 2050 emissions targets.
